# Mechanism of Unstable Material Removal Modes in Micro Cutting of Silicon Carbide

**DOI:** 10.3390/mi10100696

**Published:** 2019-10-13

**Authors:** Wei Zhao, Haibo Hong, Hongzhi Wang

**Affiliations:** 1 School of Mechanical and Electrical Engineering, Shenzhen Polytechnic, Shenzhen 518055, China; zhaowei@szpt.edu.cn; 2 Shanghai Spaceflight Precision Machinery Institute, Shanghai 201600, China; 3 Institute of Aircraft Design of AVIC Harbin Aircraft Industry Group Co., LTD., Harbin 150066, China; wang7726279@126.com

**Keywords:** ultra-precision machining, brittle-ductile cutting mode transition, molecular dynamics, undeformed chip thickness, silicon carbide

## Abstract

This study conducts large-scale molecular dynamics (MD) simulations of micro cutting of single crystal 6H silicon carbide (SiC) with up to 19 million atoms to investigate the mechanism of unstable material removal modes within the transitional range of undeformed chip thickness in which either brittle or ductile mode of cutting might occur. Under this transitional range, cracks are always formed in the cutting zone, but the stress states cannot guarantee their propagation. The cutting mode is brittle when the cracks can propagate and otherwise ductile mode cutting happens. Plunge cutting experiment is conducted to produce a taper groove on a 6H SiC wafer. There is a transitional zone between the brittle-cut and ductile-cut regions, which has a mostly smooth surface with a few brittle craters on it. This study contributes to the understanding of the detailed process of brittle-ductile cutting mode transition (BDCMT) as it shows that a transitional range can occur even for single crystals without internal defects and provides guidance for the determination of t_critical_ from taper grooves made by various techniques, e.g., to adopt larger t_critical_ around the end of the transitional range to increase machining efficiency for grinding or turning as long as the cracks do not extend below the machined surface.

## 1. Introduction

It is well known that brittle materials undergo a transition of cutting mode from brittle to ductile when the machining scale decreases to be small enough, which is referred to as brittle-ductile cutting mode transition (BDCMT) [1,2,3]. This phenomenon has been extensively studied, e.g., its mechanisms [4,5,6], how it is affected by machining conditions [7,8,9,10], etc., due to its vital importance for achieving super smooth surfaces in ultra-precision machining of brittle materials [11]. It is generally considered that the BDCMT is governed by a key parameter, i.e., the undeformed chip thickness ($t_{\mathrm{uc}}$) [12,13]. When the undeformed chip thickness is above a critical value, i.e., the so called critical undeformed chip thickness, the cutting is in the brittle mode. Otherwise ductile mode cutting would occur [14]. A number of experimental techniques have been developed to determine the critical undeformed chip thickness, e.g., fly cutting [15], plunge cutting [16], and nano-scratching [17].

While the concept of critical undeformed chip thickness has been widely acknowledged and applied to guide the ultra-precision machining of brittle materials [18,19], the cutting mode transition process around this critical value is not yet physically clear due to the lack of experimental approaches capable of observing the nanoscale cutting process in real time. A number of studies have reported that there exists a zone containing both brittle and ductile cutting behaviors between the pure ductile and brittle regions [16,20,21], but the mechanism is not yet clear. It has been suggested that this may be caused by pre-existing defects [22], or local unevenness of material properties [23], but in-depth investigations are still missing.

Molecular dynamics (MD) simulation is a prospective alternative for investigating micro scale processes [24,25], but in the past, its modelling scale was not sufficient to reproduce the BDCMT due to the lack of computation capability. The scientific computing community is significantly benefitting from the development of general-purpose computing on graphic processing units (GPU) in recent years, which can boost the computation power by up to two orders of magnitude in comparison to CPUs. Xiao et al. [26] has taken this advantage to increase the MD modelling scale to the practical machining scales in ultra-precision machining and successfully reproduced the BDCMT of single crystal silicon carbide (SiC). Therefore, MD simulation has become a viable tool for studying the ultra-precision machining processes in practical scale.

This study investigated the transitional process of cutting mode in micro cutting of single crystal 6H SiC by large-scale MD simulations with up to 19 million atoms. SiC was chosen as the workpiece due to its important applications in many industries [27,28,29], and another reason was that an advanced interaction potential capable of quantitatively reproducing its fracture behaviors was available [30]. A transitional range of undeformed chip thickness with uncertain cutting modes was observed, and the mechanisms were analyzed from the perspective of stress states in the cutting zone. The transitional range was also observed on the groove morphologies produced by a plunge cutting experiment. The findings contribute to the understanding on the detailed process of BDCMT and can be of help for understanding and optimizing the practical machining processes.

## 2. Molecular Dynamics Modelling

### 2.1. Methodology

**Figure 1:**
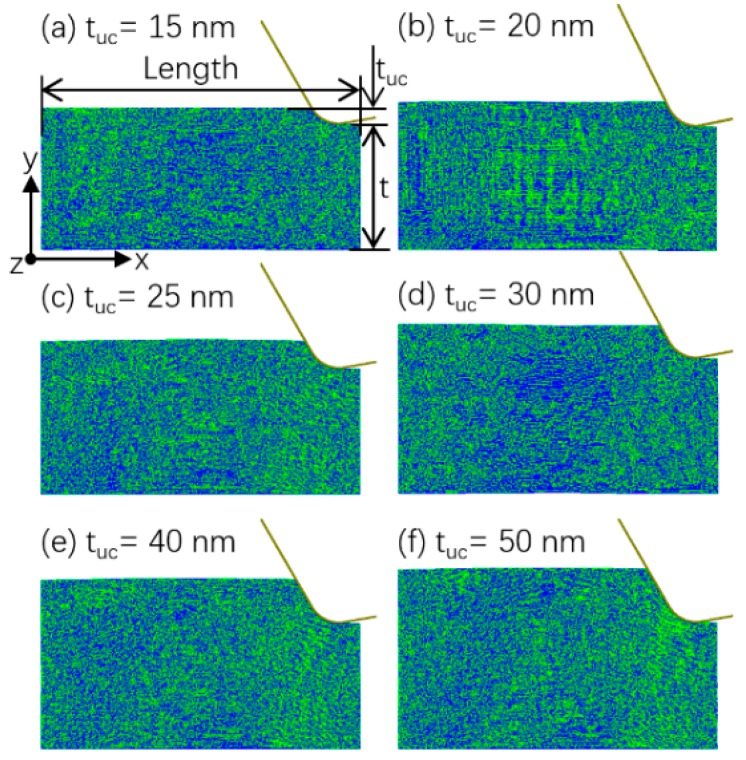
MD models of orthogonal cutting under a series of $t_{\mathrm{uc}}.$

Due to the extreme hardness of SiC, its ultra-precision machining mainly relies on abrasive processes like ultra-precision grinding and lapping [31]. The grit geometries in these abrasive processes are generally quite complicated and irregular [32]. To concentrate on the effects of undeformed chip thickness, an orthogonal cutting model was adopted in this study to simplify the geometries. This is reasonable since the machining processes of single abrasive grits in ultra-precision machining should resemble that of negatively raked cutting tools. The MD models of orthogonal cutting of 6H SiC in the (0001) <$1\bar{2}10$> orientation is shown in Figure 1. In order to save the computational time needed, the models were built in such a way that the tool was initially in contact with the workpiece material as like the cutting was already in the steady state, similar to that in ref [26]. During the simulation, the tool was moved towards the left to compress the workpiece material. The simulation was stopped when brittle fracture or ductile mode chip formation was observed. Totally six undeformed chip thicknesses were investigated, i.e., 15, 20, 25, 30, 40, and 50 nm, since the critical undeformed chip thickness of SiC was reported to be around 30 nm in this direction.

The MD codes used for the simulation were the same as those in [26], which were custom developed to implement the advanced interaction potential developed by Vashishta et al. [30] and to optimize memory utilization and computational schemes such that multi-million atom scale MD simulations could be conducted on a single GPU. The hardware utilized for the simulations was a GeForce GTX 1080 Ti with 11 GB of graphics memory from NVIDIA Corporation (Santa Clara, CA, USA). The detailed simulation parameters were summarized in Table 1. A periodic boundary was applied along the z axis, while fixed boundaries were applied in the left side and bottom of the workpiece. A thermostat region with constant temperature 20 °C was set right adjacent to the fixed boundary, so as to simulate the heat dissipation to the environment. The thickness of both the fixed boundary and thermostat region was 0.87 nm. The tool material was diamond and set as rigid body, as the cutting distance was small and tool wear should not be significant. The interaction potentials and lattice constants were the same with that in [26]. The simulations were taken out under the microcanonical ensemble (NVE). The cell size in MD simulations needs to be larger than the cut-off distance to make sure that all interactions can be included [33]. A larger cell size will increase the computation amount but at the same time will reduce the memory access via the use of shared memory when computing on GPUs. Thereby, the major concern for cell size in this study is the balance between computation and memory access, and a cell size of 0.87 nm was adopted.

The results of MD simulations are presented in the form of snapshots of atom distributions, stress distributions, and dislocation structures, since the major objective of this study is to visualize the atomistic scale details of the brittle-ductile cutting mode transition process. The codes for mechanical stress visualization and atom distribution visualization were also adopted from [26]. The dislocation structures are visualized using Visual Molecular Dynamics (VMD) (Champaign, IL, USA) [34].

### 2.2. MD Modelling Results

**Figure 2:**
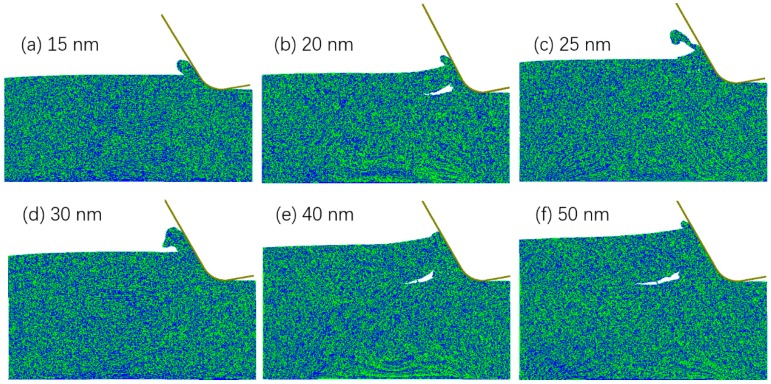
MD snapshots of the cutting mode under different $t_{\mathrm{uc}}.$

The chip formation modes for the six $t_{\mathrm{uc}}$ are shown in Figure 2. It can be seen that the cutting mode was brittle when the $t_{\mathrm{uc}}$ was 40 or 50 nm and was ductile when the $t_{\mathrm{uc}}$ was 15 nm, showing a BDCMT with the decrease of $t_{\mathrm{uc}}$. However, it is difficult to describe the relationship with cutting mode and $t_{\mathrm{uc}}$ in the range of 20 to 30 nm. Ductile mode chip formation was observed for $t_{\mathrm{uc}}$ of 25 and 30 nm, whereas mostly brittle mode chip formation was observed for the smaller $t_{\mathrm{uc}}$ of 20 nm. This indicates that the BDCMT process might not be so sharp that two different cutting modes be separated by a critical value of $t_{\mathrm{uc}}$. Instead, the brittle and ductile cutting modes were separated by a transitional range of $t_{\mathrm{uc}}$, in which both cutting modes were possible to occur.

**Figure 3:**
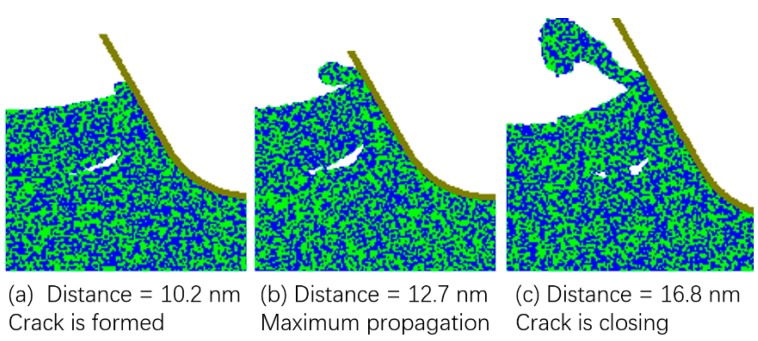
Crack formation and closing under $t_{\mathrm{uc}}$ of 25 nm.

It needs to be pointed that Figure 2 just shows the final snapshots, but the actual chip formation process for $t_{\mathrm{uc}}$ of 25 and 30 nm was more complicated. In fact, there was crack formation during the chip formation process for $t_{\mathrm{uc}}$ of both 25 and 30 nm. However, the cracks formed could not propagate and soon disappeared. Figure 3 shows the crack formation and closing under the $t_{\mathrm{uc}}$ of 25 nm. It can be seen that a small crack was formed at the cutting distance of 10.2 nm, but it did not propagate much and finally closed at the cutting distance of 16.8 nm. Here cutting distance means the distance that the tool has moved towards the left.

**Figure 4:**
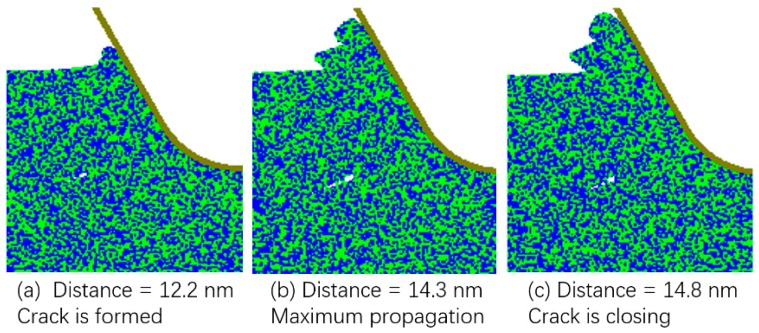
Crack formation and closing under $t_{\mathrm{uc}}$ of 30 nm.

Figure 4 shows a similar process for $t_{\mathrm{uc}}$ of 30 nm. A tiny crack was formed in the cutting zone at the cutting distance of 12.2 nm and closed at the cutting distance of 14.8 nm. This indicates that for the transitional range of $t_{\mathrm{uc}}$, cracks could always be formed in the cutting zone, but it is not sure whether they can propagate to induce brittle mode chip formation. Consequently, both brittle and ductile modes of cutting are possible under this range of $t_{\mathrm{uc}}$.

**Figure 5:**
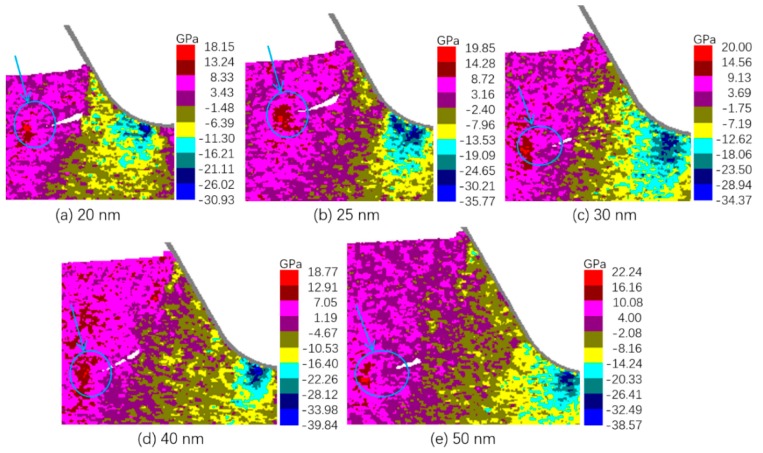
Distribution of $\sigma_{\mathrm{yy}}$ at crack formation.

In order to identify the mechanism of the unstable behavior of crack formation and propagation in the transitional range, the stress distribution was calculated. Since the observed cracks were roughly parallel to the x axis, the stress component in the y direction was analyzed. Figure 5 shows the distribution of $\sigma_{\mathrm{yy}}$ in the cutting zone when the cracks were just formed. It can be seen that a common feature of stress distribution at crack formation was that a concentration of tensile stress existed in front of the crack tip, as denoted by the cyan arrow and circle in each sub figure. It needs to be mentioned that in common sense the maximum tensile stress should exist right in the crack tip, not somewhere around it as in Figure 5. This might be due to the limitation of the algorithm for calculating mechanical stress in MD models, which is not accurate in atomically sharp corners.

**Figure 6:**
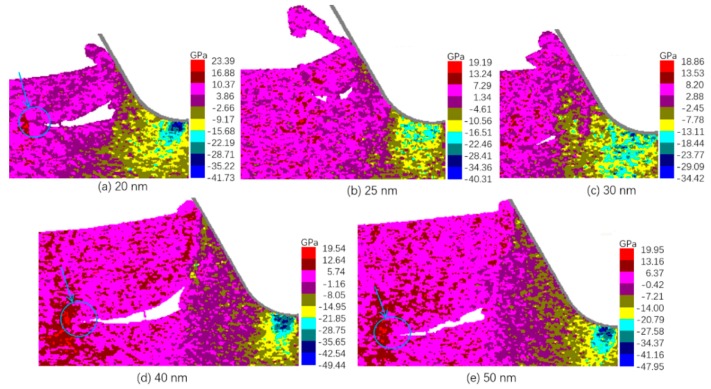
Distribution of $\sigma_{\mathrm{yy}}$ at crack propagation or closing.

The distribution of $\sigma_{\mathrm{yy}}$ during the crack propagation for $t_{\mathrm{uc}}$ of 20, 40, and 50 nm and the $\sigma_{\mathrm{yy}}$ when crack was closing for $t_{\mathrm{uc}}$ of 25 nm and 30 nm are shown in Figure 6. It is obvious that the stress concentration near the crack tip was still true for $t_{\mathrm{uc}}$ of 20, 40, and 50 nm, while no such concentration of tensile stress near the crack tip was observed for $t_{\mathrm{uc}}$ of 25 and 30 nm. This indicates that the stress states under $t_{\mathrm{uc}}$ of 25 and 30 nm did not favor the propagation of the formed cracks.

Two reasons are possible for the instability of stress states in the transitional range. One is that there is a competition between the effect of $t_{\mathrm{uc}}$ in increasing the tensile stress [26] and the effect of the negative rake face in suppressing the crack propagation [4]. When the $t_{\mathrm{uc}}$ increases, the contact area between rake face and workpiece increases, and thereby, the projected contact area downwards also increases, which is expected to increase the crack suppressing effect. In this transitional range of $t_{\mathrm{uc}}$, the delicate balance between the two opposite effects might be broken towards different sides, leading to uncertainty in crack propagation or closing. Another is that there might be a certain level of randomness in the location of crack initiation under a given $t_{\mathrm{uc}}$. The location of crack initiation could affect the stress distribution afterwards and thereby add some uncertainty to the subsequent propagation.

**Figure 7:**
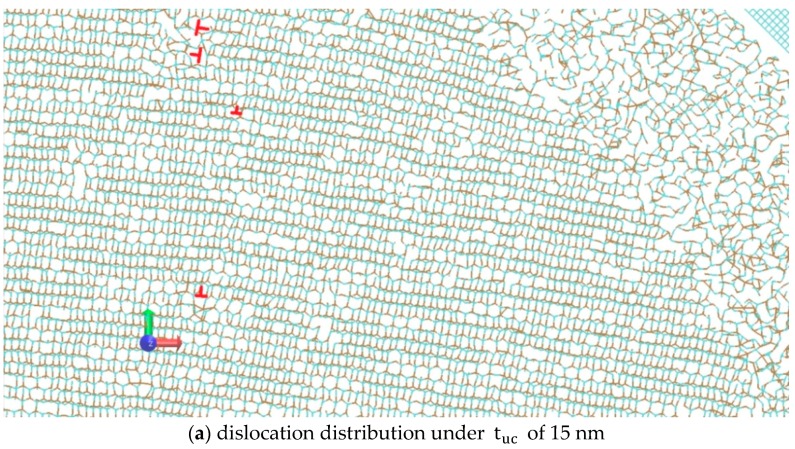

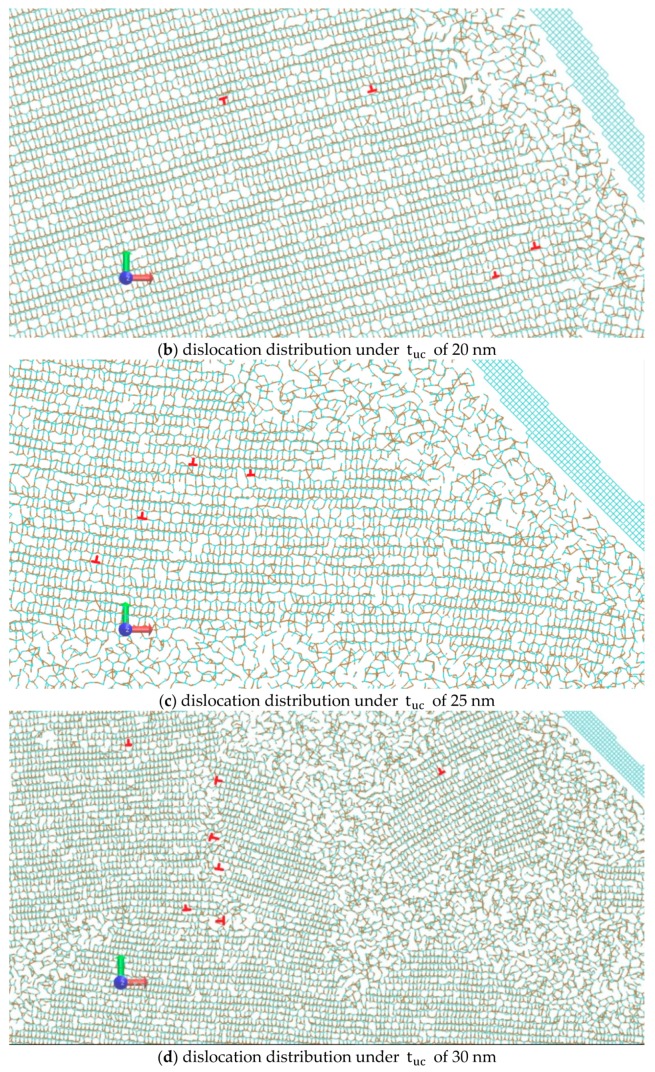
Dislocations in the machining zone under several $t_{\mathrm{uc}}.$

Figure 7 shows the dislocations in the machining zone using red ‘⊥’ symbols under the $t_{\mathrm{uc}}$ of 15, 20, 25, and 30 nm, which were visualized using the VMD software [34]. The chip formation was mainly via brittle fracture under the $t_{\mathrm{uc}}$ of 40 and 50 nm, and dislocations were quite rare. A common feature of Figure 7a–d is that there exists a highly dislocated zone right in front of the tool rake face, which might be due to the high compressive stress in this region. The lattice structure is less disordered in the zones next to this highly dislocated zone, and dislocations can be identified. It can be seen that the dislocations are mainly basal plane edge dislocations on the basal plane slip system and Frank partial dislocations. The Burgers vector of the basal plane edge dislocations is a/3<$1\bar{2}10$>, while the Burgers vector of the Frank partials is along the c-axis of 6H SiC. Their resultant movement can lead to the upwards flow of workpiece material along the cutting direction.

## 3. Experimental Validation

A plunge cutting experiment was conducted to verify the MD modelling results. The experimental setup is described in ref [35]. The workpiece was cut out from a piece of (0001) 6H SiC wafer from Powerway Advanced Material Company (Xiamen, China). A single crystal diamond tool from Apex Diamonds (Pittsburgh, PA, USA) was used for the experiment. The major experimental parameters were summarized in Table 2.

**Figure 8:**
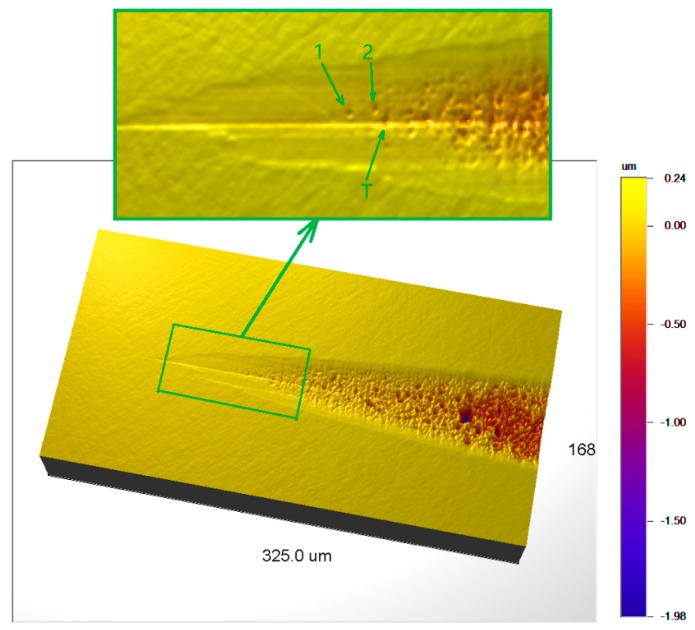
Groove morphology by plunge cutting.

The plunge cutting produced a taper groove as shown in Figure 8, which was measured using a white light interferometer [36,37]. Distinct difference between ductile-cut and brittle-cut surfaces can be observed. An enlarged view of the transition zone is shown in the upper part of Figure 8, as denoted by the green rectangle. Between the ductile-cut and brittle-cut zones, several small craters can be observed, e.g., those marked by ‘1’, ‘2’, and ‘T’. It can be seen that the surface around these craters is still very smooth, indicating ductile mode of material removal. This means that for the range of $t_{\mathrm{uc}}$ around these craters, both ductile and brittle mode cutting are possible, which is in good agreement with the MD results.

**Figure 9:**
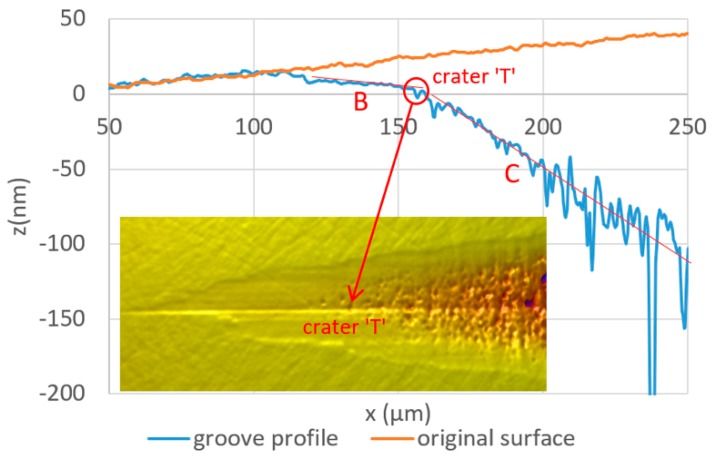
Groove profile along the centre line.

Figure 9 shows the profile of the groove in the center line. There is a significant difference in the surface slopes of the ductile-cut and brittle-cut regions, as denoted as ‘B’ and ‘C’ respectively. Two red lines are drawn in Figure 9 to represent the average slopes of the two regions. Their intersection point is roughly located at the point following the crater ‘T’. Therefore, it is considered that the transitional range ends after crater ‘T’.

**Figure 10:**
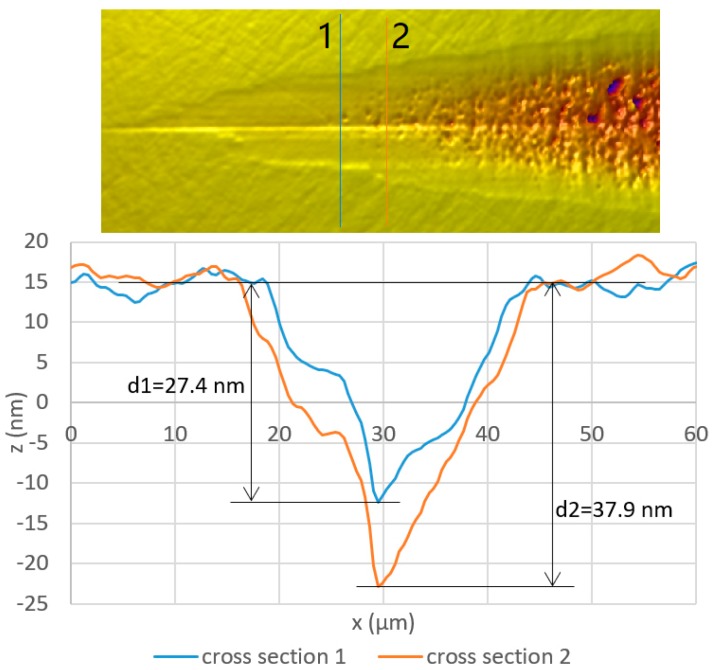
Cross-sectional profiles start and end of transitional range.

The cross-sectional profiles of the taper groove at the start and end points of the transitional range are shown in Figure 10. The depth of the groove at these two points are measured to be 27.4 and 37.9 nm, respectively, indicating a transitional range of $t_{\mathrm{uc}}$ between 27.4 and 37.9 nm. In comparison, the transitional range of $t_{\mathrm{uc}}$ in the MD simulations was from 20 to 30 nm. It is encouraging that the quantitative values of the transitional range from the MD simulations and experiment are on the same scale, considering that the MD simulation results are affected by many factors, e.g., the quality of the interaction potential for quantitatively reproducing the mechanical properties, the simplification of the machining model, and the difference in cutting speeds, etc. The important thing is that both the simulations and experiment showed that there exists a transitional range of undeformed chip thickness during which both ductile and brittle mode material removal might occur.

The transitional range of undeformed chip thickness explains why scattered tiny craters existed between the ductile-cut and brittle-cut regions in many studies [16,20,21]. It reveals that these scattered craters could occur even if there are no disturbances like pre-existing defects, local unevenness, vibrations in machining system, etc. This can serve as guidance for determining the “critical undeformed chip thickness” from the taper grooves or scratches made by various experimental techniques. If the machining process tolerates no brittle fracture, the “critical undeformed chip thickness” should be selected at the point before the start of the transitional range. A larger “critical undeformed chip thickness” at somewhere before the end of the transitional range can be adopted if the machining process accepts minor brittle fractures as long as they do not extend below the machined surface, providing higher machining efficiency.

It needs to be mentioned that the experimental cutting speed of 3 mm/s was much lower than that in the MD simulations, i.e., 100 m/s. Two factors need to be considered regarding the effect of cutting speed on the results. One is the heat effect which might affect the mechanical properties of the workpiece material. Though the cutting speed in the MD simulation was quite high, the total time of simulation was very short, and the heat accumulation would not be significant. Thereby it is supposed that the high cutting speed in MD simulation would not bring much heat softening of the workpiece material. Another factor is the shock wave which might be induced by the high cutting speed. It was reported that the shock wave speed in cubic SiC was ~11.5 km/s, and amorphization would occur when the impact speed exceeded 4.91 km/s [38]. The cutting speed in the MD simulation was much lower than this critical value, and at the same time, the cutting distance was quite short, i.e., ~20 nm, when compared to the length of workpiece, i.e., 300 nm. It is expected that the shock wave induced by the impact of the cutting tool would not have much influence on the mechanical properties of the workpiece material.

## 4. Conclusions

The mechanism of unstable material removal modes in micro cutting of 6H SiC was investigated by large scale MD simulations and plunge cutting experiment. It is found that the brittle and ductile cutting modes were not simply separated by a critical undeformed chip thickness. Instead, a transitional range of undeformed chip thickness with uncertain cutting modes was observed, though there are no internal defects in the single crystal. The MD simulations showed that cracks were always formed in the cutting zone under this transitional range of undeformed chip thickness. However, the stress states in the cutting zone were not always sufficient to propagate the cracks. The cutting mode was in brittle when the cracks did propagate and otherwise in ductile. This provides guidance for interpreting the surface morphologies produced by various experimental techniques for studying the BDCMT behaviors, and helps optimizing the machining parameters for ultra-precision grinding or cutting of brittle materials, e.g., increase undeformed chip thickness to the end of the transitional range if the cracks do not extend below the machined surface.

## Figures and Tables

**Table 1:** MD Simulation parameters of orthogonal cutting.

Parameter	Value
Length of workpiece	300 nm
Thickness	3.5 nm
t	120 nm
Undeformed chip thickness	15, 20, 25, 30, 40, 50 nm
Atoms in workpiece	11.8, 13.0, 14.2, 14.9, 17.9, 18.7 million
Total atoms in Tool	0.28 million
Tool rake angle	−30°
Tool clearance angle	10°
Cutting edge radius	30 nm
Cutting speed	0 m/s

**Table 2:** Parameters in plunge cutting experiment.

Parameter	Value
Tool rake angle	−30°
Clearance angle	10°
Tool nose radius	1.507 mm
Cutting speed	3 mm/s
Tilt angle	0.01°
